# T lymphocytes need less than 3 min to discriminate between peptide MHCs with similar TCR-binding parameters

**DOI:** 10.1002/eji.201445214

**Published:** 2015-04-21

**Authors:** Alexandre Brodovitch, Eugene Shenderov, Vincenzo Cerundolo, Pierre Bongrand, Anne Pierres, Philip Anton van der Merwe

**Affiliations:** 1 Lab Adhesion Cellulaire and Inflammation, Aix-Marseille UniversitéFrance; 2 INSERM U1067France; 3 CNRSU7333, France; 4 MRC Human Immunology Unit, Weatherall Institute for Molecular Medicine, University of OxfordOxford, UK; 5 Assistance Publique, Hôpitaux de MarseilleFrance; 6 Sir William Dunn School of Pathology, University of OxfordOxford, UK

**Keywords:** Affinity, Early T-cell activation, Interference reflection microscopy, Kinetics, pMHC, Spreading, TCR

## Abstract

T lymphocytes need to detect rare cognate foreign peptides among numerous foreign and self-peptides. This discrimination seems to be based on the kinetics of TCRs binding to their peptide–MHC (pMHC) ligands, but there is little direct information on the minimum time required for processing elementary signaling events and deciding to initiate activation. Here, we used interference reflection microscopy to study the early interaction between transfected human Jurkat T cells expressing the 1G4 TCR and surfaces coated with five different pMHC ligands of 1G4. The pMHC concentration required for inducing 50% maximal IFN-γ production by T cells, and 1G4-pMHC dissociation rates measured in soluble phase or on surface-bound molecules, displayed six- to sevenfold variation among pMHCs. When T cells were dropped onto pMHC-coated surfaces, rapid spreading occurred after a 2-min lag. The initial spreading rate measured during the first 45 s, and the contact area, were strongly dependent on the encountered TCR ligand. However, the lag duration did not significantly depend on encountered ligand. In addition, spreading appeared to be an all-or-none process, and the fraction of spreading cells was tightly correlated to the spreading rate and spreading area. Thus, T cells can discriminate between fairly similar TCR ligands within 2 min.

## Introduction

An essential step of adaptive immune responses is the recognition by T lymphocytes of a cognate antigen exposed as a peptide–MHC (pMHC) complex on an antigen-presenting cell (APC). This recognition possesses remarkable sensitivity, speed, and selectivity 1. Indeed, some T cells can detect a few and perhaps a single pMHC 2–4. T-cell/APC encounters may trigger a signaling event, such as phosphorylation or transient calcium rise, within a few seconds 5 and a physiological response, such as arrest 6 or spreading 7, within minutes. The specificity of adaptive responses requires that a T cell is able to discriminate between a few cognate pMHCs and nearly 10 000-fold excess of self pMHCs. While the mechanisms of signal triggering are not fully elucidated, the current view is that TCR-mediated pMHC discrimination relies on quantitative properties of the TCR/pMHC interaction rather than structural differences in the binding interface 8,9. Indeed it is well-established that the capacity of pMHC to stimulate a TCR correlates with the affinity and even more strikingly the lifetime 10–14 of TCR/pMHC interaction as measured using soluble molecules, that is, under so-called 3D conditions, which are comparable to lifetimes measured on molecules bound to inert surfaces 15. Recent reports suggest that molecular interactions occurring between cell-bound TCRs and ligands behave somewhat differently from 3D interactions 16,17, and the force dependence of TCR-pMHC interactions is an important factor 18. Nevertheless, the accumulated evidence strongly suggests that T cells can discriminate between pMHCs that bind their TCR with intrinsic lifetimes differing by a factor lower than 10, and those shorter lifetimes cannot necessarily be compensated by increases in pMHC concentration.

It has long been recognized that fundamental physical principles impose a limitation on the specificity of biomolecule interactions 19,20. Indeed, since bond rupture is a random event, determining the lifetime of a single TCR/pMHC interaction cannot yield accurate information on the precise dissociation rate. A possible way for a TCR to overcome this limitation is to perform multiple measurements. Sensitivity can be retained if measurements are performed on the same pMHC due to serial rebinding 21, but this will result in a trade-off between specificity and speed 14,22. Indeed, if the intrinsic lifetime of interactions between a TCR and cognate pMHC is of the order of 10 s 10, performing multiple lifetime determinations on a single TCR/pMHC couple may require a minute or more. One way to overcome this limitation is to apply forces on TCR/pMHC bonds, thus reducing their lifetime and enhancing discrimination 22,23, but this may be difficult to reconcile with recent results, suggesting that productive TCR/pMHC interactions display an unusual resistance to forces 18.

Thus, there is a strong need for an accurate determination of the minimum time required by a T lymphocyte to discriminate between pMHC complexes that bind to its TCR with different affinities and lifetimes. Previous studies of the T lymphocyte capacity to discriminate between different pMHCs relied on responses such as target cytolysis or cytokine release several hours after initial stimulation 4,10,13. The present report was aimed at looking for the minimal time required by T cells to discriminate between pMHCs bound by their TCR with fairly similar strength. We took advantage of the finding that an early reporter of the cell decision to enter an activation program is an active and rapid spreading on surfaces exposing TCR ligands 7,24,25. We used a recently described 7,24,25 implementation of interference reflection microscopy (IRM) to achieve real-time quantification of the earliest step of contact formation between Jurkat cells expressing the 1G4 TCR and planar surfaces presenting a series of five pMHC ligands. These ligands have been shown to bind the 1G4 TCR with lifetimes spanning a sevenfold range as measured under 2D 15 and 3D 13 conditions, which correlated with their capacity to trigger interferon-γ production 13. We show that cells falling on activating surfaces establish transient contacts of about 6 s during a lag period in the order of 2 min before displaying a robust spreading. In contrast with the lag period duration, both spreading rate and maximum contact area were strongly dependent on the peptide exposed by the surface. Peptides with higher activation efficiency triggered a higher spreading rate and a greater spread area. Thus, T cells were able to discriminate between highly similar pMHCs within a few minutes.

## Results

### T-cell contact with a surface bearing TCR ligands induces an all or none response within 3 min

**Figure 1:**
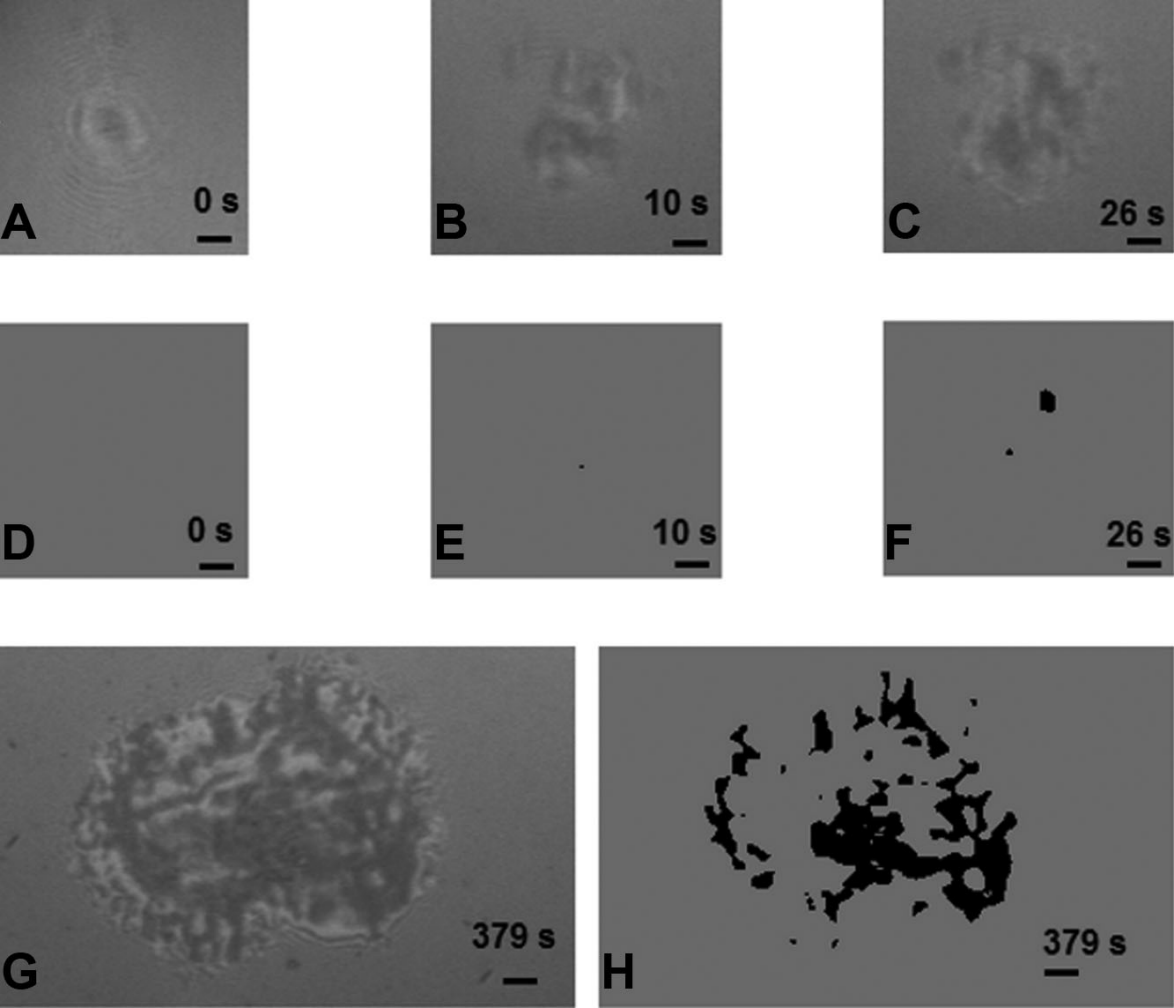
Typical images of spreading Jurkat cells on pMHC-coated surfaces. 1G4-transfected Jurkat T cells were sedimented onto pMHC-coated surfaces under microscopic observation and recording (One image per second). (A to C, G) Sequential IRM images of a typical cell. (D to F, H) Computer-calculated contact areas (shown as black pixels on a gray area). (A) Initial aspect of sedimenting cells with concentric rings indicative of a fairly spherical shape. (B and E) Point-like contact (0.03 μm^2^) that appeared 10 s later. (C and F) Display two contact spots with a total area of 1.1 μm^2^ (time = 26 s). (G and H) Represent a more extensive contact (4150 pixels corresponding to 5.9 μm^2^; time = 379 s). Bar = 2 μm. Images are representative of 24 independent experiments performed, with about 20 studied individual cells per experiment.

CD8-negative Jurkat T cells bearing 1G4 TCR were injected into a chamber coated with pMHCs and a random field was monitored with IRM for at least 5 min. Images were recorded and processed for contact area determination. Typical images are displayed in Figure 1: cells first appeared as fairly circular structures with concentric rings, in accordance with the expected image of spherical objects in suspension (Fig. 1A). They displayed typical Brownian motion, with a minimum calculated distance to the surface ranging between 40 and 70 nm. When pMHCs were 1G4 TCR ligands, a fraction of cells ranging between 26 and 81% stopped and formed contacts with the substratum (Fig. 1B and C). After a variable lag, the contact area exhibited a rapid increase and reached a maximum of 10–100 μm^2^ (Fig. 1G and H) within 2–3 min. A slow decrease of the contact area followed during the next tens of minutes. A representative movie is shown (see Supporting Information). As expected, no spreading response was observed when 1G4 ligand was replaced with an irrelevant pMHC complex (not shown).

### T-cell spreading is preceded by an observation period involving transient cell to surface contacts

**Figure 2:**
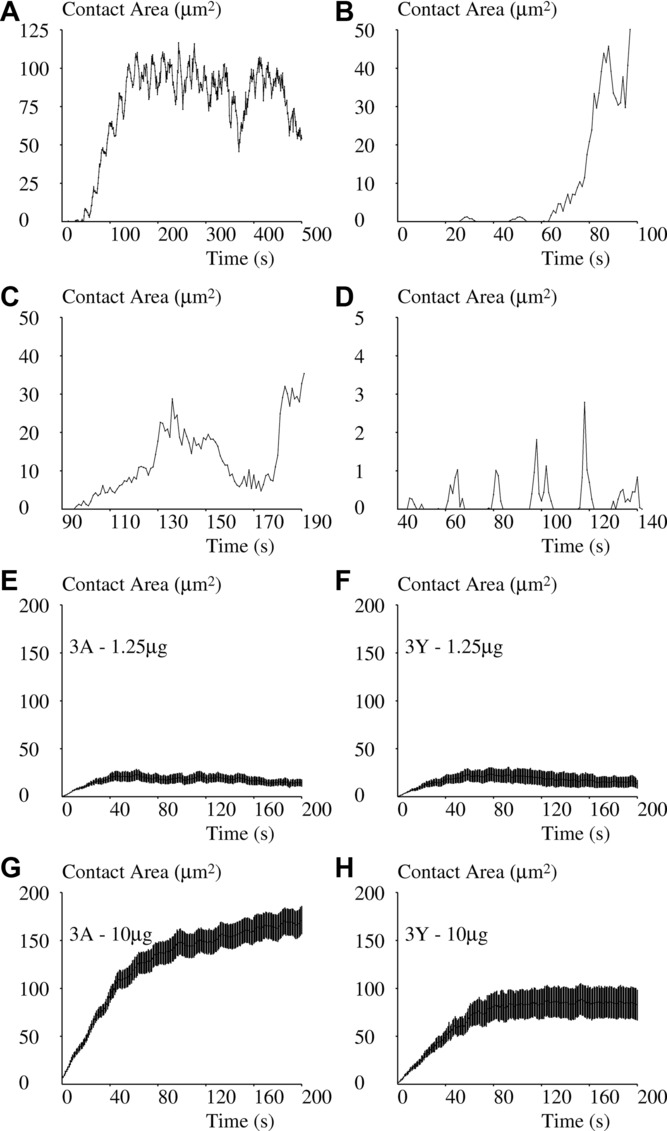
Spreading of 1G4-transfected Jurkat T cells onto pMHC-coated surfaces as plotted over time. (A–H) 1G4-transfected Jurkat T cells were sedimented onto surfaces coated with 1G4 ligand under IRM observation and recording (1 image/s). A total of 495 individual cells were followed for 10 min each and images were processed to build contact plots. (A and B) The most frequently recorded curves with rapid spreading frequently preceded with transient contacts are shown. (C) A less clear-cut spreading pattern with delayed appearance of a rapid spreading phase is shown. (D) A fairly rare case with repeated transient contacts without extensive spreading is shown. (E and F) The average spreading of cells on a surface coated with a lower amount (1 μg) of peptide bound by their TCR with (E) a lower dissociation rate (3A, 35 cells) or (F) a higher dissociation rate (3Y, 30 cells) is shown. (G, H) The average spreading of cells on a surface coated with a higher amount (10 μg) of (G) a more strongly bound peptide (3A, 50 cells) or (H) a less strongly bound peptide (3Y, 26 cells) are shown. (E to H) Data are shown as mean ± SEM of about 20 individual cells observed in 5 independent experiments for each condition (defined as a pMHC species and a concentration).

**Figure 3:**
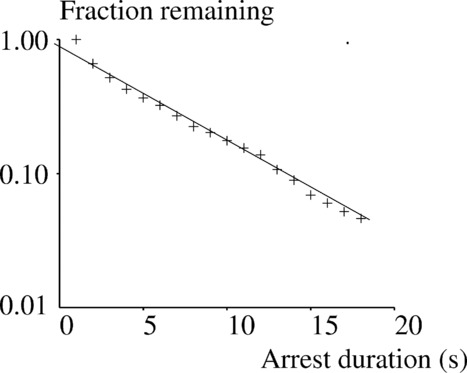
Frequency distribution of transient contacts. The frequency distribution of transient contacts such as exemplified in Figure 2B is represented as a survival plot revealing a typical power law with an exponent of −0.19. The curve was built out of 345 recorded values of contact duration. Data are representative of 24 independent experiments.

The spreading kinetics of a total of 495 cells was obtained on surfaces presenting a total number of five pMHC species and four surface concentrations each. This observation revealed the following behavioral patterns, as exemplified in Figure 2:

- The most frequent response (27% of cases) consisted of a rapid increase of contact area with a spreading rate on the order of several micrometer square per second (Fig. 2A). Interestingly, small area fluctuations with a period in the order of 10 s were frequently visible on the ascending part.
- In about 63% of these cells, the robust spreading response was preceded by several (mean: 3.8 per cell) transient contacts (Fig. 2B) with a mean duration of 5.9 s ± 9.2 SD (*n* = 345 arrests). The distribution of these transient contact durations followed a power law with an exponent of –0.19 (Fig. 3).
- Ten percent of cells displayed a less clear-cut response with delayed (Fig. 2C) or no definitive spreading (Fig. 2D).
- Average responses of cells deposited on lower (Fig. 2E and F) or higher (Fig. 2G and H) concentrations of more active (3A, Fig. 2E and G) or less active (3Y, Fig. 2F and H) pMHC are also shown.

### Cellular spreading is strongly influenced by the quality of pMHCs

First, the contact area measured 15–20 min after cell deposition was on surfaces coated with five different pMHC species. As summarized in Table 1, the contact area displayed 13-fold variation when the pMHC was varied, thus showing that cells efficiently discriminated between these TCR ligands within 20 min.

**Table 1:** Relationship between pMHC and T-cell spreadinga)

Peptide	EC50 (μg/mL)	3D *k*_off_ (s^−1^)	2D *k*_off_ (s^−1^)	Contact area (μm^2^)	Initial spreading rate (μm^2^/s)	Maximum spreading rate (μm^2^/s)	Lag before max spreading (s)
3A	70	0.11	0.076	62.7 ± 0.011 SEM	2.13 ± 0.23 SD	2.58 ± 0.23 SEM	130 ± 19 SEM
H74	107	0.13	0.133	40.1 ± 0.011 SEM	2.04 ± 0.50 SD	3.02 ± 0.43 SEM	73 ± 18 SEM
9V	180	0.09	0.271	70.2 ± 0.013 SEM	2.23 ± 0.14 SD	2.64 ± 0.06 SEM	75 ± 5 SEM
3Y	240	0.61	0.477	13.6 ± 0.007 SEM	1.22 ± 0.22 SD	1.70 ± 0.07 SEM	120 ±7 SEM
9L	426	0.37	0.512	4.9 ± 0.005 SEM	1.24 ± 0.12 SD	1.52 ± 0.28 SEM	90 ± 33 SEM
Significance				*p* < 10^−10^	*p* < 10^−10^	*p* = 0.067	*p* = 0.21

Transfected Jurkat T cells bearing 1G4 TCR were deposited on surfaces pretreated with different pMHCs and assayed for spreading at the single-cell level. The activation potency was expressed as the pMHC concentration for 50% maximal stimulation of interferon production 13, 3D *k*_off_ was obtained with Biacore 13 and was obtained with a flow chamber operated on molecules bound to inert artificial surfaces [15]. Contact area was measured between 15 and 20 min after cell deposition. Initial spreading moment was determined by visual examination of spreading curves and calculation of the average slope was done on the time period of 45 s following rapid contact extension. The maximum spreading rate and the time period between initial contact and beginning of the 45-s period with maximum spreading rate were calculated on a subpopulation of cells that could be observed before any contact formation with the surface. The significance of difference between peptides was calculated with analysis of variance and Satterwaith correction for unequal samples.

**Figure 4:**
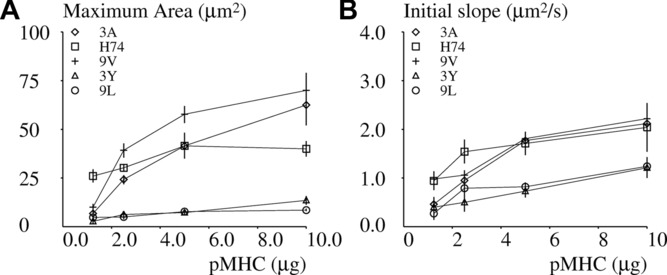
Dependence of spreading on deposited peptide-MHC. 1G4-transfected Jurkat cells were deposited on surfaces coated with varying amounts of peptide-MHC. 3A (diamond), H74 (square), 9V (crosses), 3Y (triangles), or 9L (circles) and the average spreading area was determined in the period of time ranging between 15 and 20 min after deposition. (A) Each point represents a mean of 249–1096 values. (B) Each point represents the average spreading rate of cells that displayed an active response. (A and B) A total number of 495 cells were studied. Vertical bar length = twice the SD. Data shown are representative of 24 independent experiments.

The dependence of spreading on pMHC concentration was also studied: As shown in Figure 4, results allowed clear-cut discrimination between 2 groups of pMHCs. Thus, the pMHCs 3A, H74, and 9V yielded a higher contact area, even after a fourfold dilution, than 3Y and 9L. They also displayed a higher capacity to stimulate interferon-γ production, and formed more durable bonds with 1G4 TCR, as measured under both 3D and 2D conditions (Table 1). However, no single binding or spreading parameter was fully correlated with interferon-γ production, thus excluding the simple hypothesis that the initial discrimination between different pMHCs fully determines T-cell behavior for several hours following initial antigen detection.

### Peptide discrimination is clearly visible within 2 min after initial stimulation

It was important to determine whether the 15- to 20-min period between initial encounter and contact area measurement was required to allow peptide discrimination. We addressed this question by monitoring the beginning of contact formation on individual cells. As shown in Table 1, the pMHCs that induced higher contact areas also induced more rapid initial spreading, although the initial spreading rate displayed only 1.8-fold variation depending on stimulation peptide, but peptide discrimination remained highly significant (*p* = 1.3 × 10^−16^). As shown in Figure 4B, this difference was fairly robust with respect to peptide dilution.

**Figure 5:**
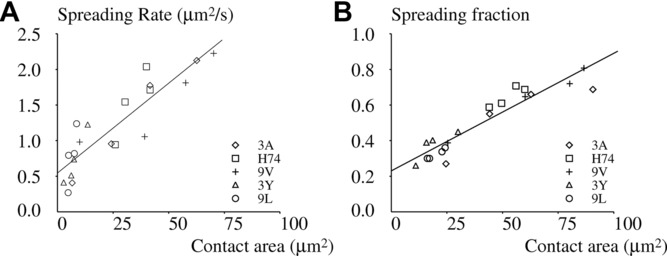
Spreading rate, contact area, and spreading fraction are tightly correlated. 1G4-transfected Jurkat cells were deposited on surfaces coated with varying amounts of MHC-coupled peptide 3A (diamond), H74 (square), 9V (crosses), 3Y (triangles), or 9L (circles). The fraction of cells with a spreading response (13 574 cells), the contact area of cells that displayed a spreading response (6622 cells), and the initial spreading rate of cells that displayed a spreading response were measured. (A) The correlation coefficient between spreading rate and contact area was 0.8915 and (B) the correlation coefficient between the spreading fraction and the contact area was 0.9362. Data shown are representative of 24 independent experiments, with an average of 20 samples/group.

It was important to assess whether the initial spreading rate and later contact area were determined by similar criteria. As shown in Figure 5A, when pMHC structure and concentration were varied independently, both parameters where highly correlated (*r* = 0.8915), suggesting that substratum analysis was fully completed at the moment that spreading initiated.

We checked this hypothesis by studying the fraction of cells that displayed a spreading response. As shown in Figure 5B, the variations of spreading fraction closely matched spreading area (as derived from Fig. 4A), with both parameters tightly correlated (*r* = 0.9362).

### Peptide analysis occurs within 2 min, and this duration weakly depends on pMHC specificity

**Figure 6:**
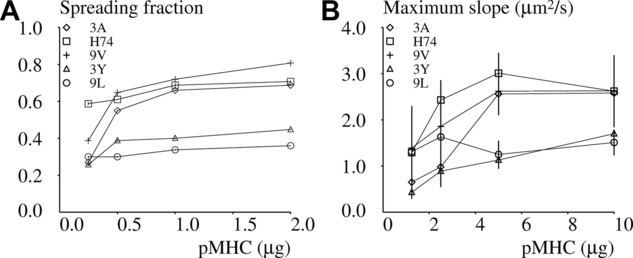
Dependence of spreading fraction and maximum slope on activating surfaces. 1G4-transfected Jurkat cells were deposited on surfaces coated with varying amounts of the 3A (diamond), H74 (square), 9V (crosses), 3Y (triangles), or 9L (circles) pMHCs. (A) The fraction of cells that displayed an active spreading response was determined. (B) The maximum spreading rate was determined in the period of time ranging between 0 and 10 min after deposition. A total number of 297 cells that could be monitored for 10 min and that did not display any contact at time zero were studied. (A and B) Vertical bar length = twice the SEM. Data shown are representative of 24 independent experiments, with an average of 20 samples/group.

**Figure 7:**
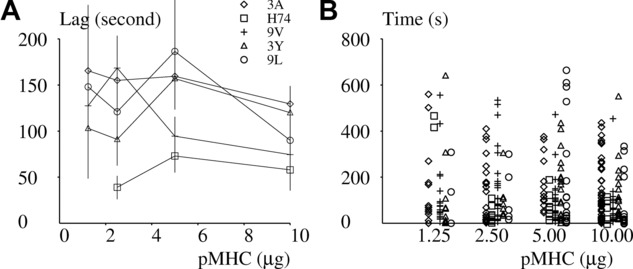
Relationship between surface coating and duration of analysis. 1G4-transfected Jurkat cells were deposited on surfaces coated with varying amounts of the 3A (diamond), H74 (square), 9V (crosses), 3Y (triangles), or 9L (circles) pMHCs and the duration of the lag between initial contact and beginning of spreading at maximal rate was determined on 297 cells that displayed a spreading response and were not in contact with the surface at the onset of the observation period. (A) Average value of the lag between initial contact and spreading at maximum rate. Vertical bar length = twice the SEM. (B) Individual values of the lag between initial contact and spreading at the maximum rate. Data shown are representative of 24 independent experiments, with an average of 20 samples/group.

In order to obtain more information on the duration of the analysis period, we used a fully automatic method to determine the length of the delay between initial contact and starting point of the 45-s period with maximum spreading rate. The analysis was restricted to 297 individual cells (out of 495) that were not in contact with the surface at the beginning of video recording. The following observations were made:

- As shown in Table 1 and Figures 4B and 6B, the maximum spreading rate correlated with the initial spreading rate, and the separation of the pMHCs into two groups, with higher (3A, H74, and 9V) and lower (3Y and 9L) activation efficiency, was confirmed.
- As shown in Table 1 and Figure 7, the lag between initial cell to surface contact and spreading “burst” did not display the same dependence on pMHC and pMHC concentration as spreading rate and spreading area; in accordance with the hypothesis, the lag represents a period of information acquisition and processing. When pooling data obtained on all pMHCs, the average lag was, respectively, 108.6 ± 9.9 s SEM (*n* = 101 values), 136.3 ± 16.3 s (*n* = 88), 132.9 ± 17.4 (*n* = 62), and 146.2 ± 26.1 (*n* = 46) when surfaces were coated with 10, 5, 2.5, and 1.25 μg pMHC.

The surface density σ of pMHCs was determined by labeling with fluorescent anti-HLA and fluorescence determination. It was found that:

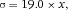
1where σ is in molecule per micrometer square and *x* is the amount of pMHC in microgram.

## Discussion

The purpose of this work was to determine the minimum time needed by T cells to discriminate between two slightly different pMHC ligands. We used cell spreading as an early and robust reporter of the cell decision to initiate an activation program 7,24,25. Our results show that clear-cut discrimination between pMHCs agonists that display only quantitative differences in their capacity to induce interferon-γ production 13 is completed within a 2-minute period preceding the spreading burst. The probability (fraction of responding cells) and intensity (initial spreading rate and maximum spreading area) were highly correlated, suggesting that they were part of a same cell decision and functional program.

There are some key advantages to using IRM to monitor cell-surface contacts. First, it allows clear-cut detection of effective molecular contacts. Studies done with electron microscopy have long shown that the apparent contact area revealed by conventional light microscopy between lymphocytes and their targets markedly exceeds the true contact area 27. Second, it is not necessary to label the cells with fluorescent probes or illuminate them with intense light, reducing the likelihood of artifacts. Third, the range of IRM analysis is ideally suited to monitor molecular contacts. The maximum contrast is seen when membrane-to-surface gap varies between about 0 and 100 nm, and the length of typical ligand–receptor couples ranges between about 14 nm (e.g., TCR-pMHC, CD2-CD58, or CD28/CD80-CD86) and 40 nm (LFA-1-ICAM1) or even 80 nm (P-selectin-PSGL-1). The dominant glycocalyx molecules, such as CD43 or CD45, are approximately 30–50 nm.

While calculating the distance using Equation (2) is an approximation 28,29, the cell surface is too complex to warrant more detailed analysis. Furthermore, as previously emphasized 26,30, Equation (2) is fairly robust and calculations are not affected by image processing, such as background subtraction or contrast enhancement. Finally, the threshold we used for defining cell-surface contact could be validated by checking that the occurrence of a dark zone with subthreshold darkness was correlated with the resistance to flow-induced detachment 26 and a decrease in Brownian motion (not shown).

The cells used in this experiment lack expression of the CD8 coreceptor. It is likely that the presence of CD8 would greatly increase the sensitivity of pMHC detection 31, and it has been argued that a requirement for CD8 or CD4 engagement may further enhance the ability of T cells to discriminate between pMHCs 32. We chose to exclude CD8 to facilitate direct comparison between TCR/pMHC binding parameters 13,15 and cell responses and test intrinsic TCR-mediated recognition. What we showed here is that the TCR has an intrinsic capacity to generate different messages during the first few minutes following encounter with fairly similar ligands that are bound with roughly comparable “strength.” This is likely to contribute the difference between functional outcomes such as cytokine release or proliferation, which are generated during the following hours as a consequence of multiple and highly complex biochemical signals. Coreceptors such as CD8 would influence both binding and signaling events, starting from a very early phase 8. Future experiments will examine the contribution of the CD8 coreceptor.

Another point illustrates the complexity of involved mechanisms and may warrant the interest in a simplified (CD8-free) model system: As shown in Table 1 and Figure 4, even the early spreading response could not be accounted for by a single signal. Thus, the dose-dependence of spreading rate and area is clearly different between H74 and other peptides. Also, the spreading area, lag time before spreading, and dissociation rates are not ordered in a monotonous way (Table 1). This strongly suggests that spreading is not driven by a sequence of clear-cut events, but is likely to be determined by a network of events. An attractive hypothesis might be that this complexity is due to the high level of performance required by T lymphocyte activation.

The nature of surface scanning behavior is markedly dependent on cell status. Recent TIRF experiments suggest that human peripheral blood lymphocytes use filopod-like protrusions to probe surfaces 25,33, in accordance with the previous hypothesis that filopodia are directly involved in environment probing 34,35. TIRF experiments performed on cells used for the present study (not shown) revealed different contacts associated with deformations of the whole cell membrane that were much more difficult to analyze. These differences motivated the choice of IRM for the present study.

An advantage of our approach is that it provides information at the single-cell level. Indeed, it may be difficult to derive information on cell function from results obtained on bulk populations 36 since a graded response at the population level may be a consequence of all or none (or digital) response at the single cell level with varying threshold. Our result suggest that cell decision to spread is digital, as seen on inspection of spreading curves (Fig. 2), whereas the average contact area and spreading rate constitute analogue responses that vary according to cell stimulation. Interestingly, the spreading fraction is also quantitatively related to the final contact area of cells that have decided to spread (*r* = 9362, Fig. 6B).

A long-standing difficulty in understanding the specificity of T cell recognition is to reconcile the hypothesis that T-cell activation is determined by the lifetime of TCR-pMHC bond and the finding that widely different responses are induced by pMHCs that are bound with dissociation rates differing by a factor lower than 10. Possible ways of resolving this paradox were the so-called proofreading mechanism 20, with a need for a TCR/pMHC interaction to be sufficiently long to be productive, or the rapid summation of multiple interactions 22, or the hypothesis that forces generated at the T-cell/APC interface might increase the difference between bond lifetime involving agonist and antagonist pMHCs 16–18. The (nonexclusive) mechanisms suggested by our results would be that T cells might sum the TCR–pMHC interaction occurring during a fairly fixed period of time of about 2 min and take a decision accordingly. This period is consistent with the results of Liu et al. 18 and matches well the order of magnitude of the duration of TCR/APC interaction occurring in lymph nodes under physiologic conditions 37,38. Our experimental model provides a suitable tool for studying the possible mechanism of this putative summation process.

## Materials and methods

### Molecules and surfaces

Experimental procedures were as previously described 13,15. Briefly, pMHCs were HLA-A2 molecules in complex with the peptide SLLMWITQV (9V) and variants thereof that differed by a single amino acid in the peptide (3A, 3Y, 9L) or the MHC (H74). Glass surfaces (1 cm^2^) were cleaned with a mix of 70% sulfuric acid and 30% H_2_O_2_, then rinsed thoroughly and coated with poly-l-lysine (150 000—300 000 Da), then incubated in glutaraldehyde for coating with biotinylated BSA. After blocking unreacted aldehyde groups with glycine, slides were coated with neutravidin before adding different amounts of biotinylated pMHCs. The surface density of pMHCs was determined by labeling with an excess of Alexa Fluor 488 labeled anti-HLA antibody (#311415, Biolegend, San Diego) and fluorescence determination. Absolute calibration was done as previously described 39 by measuring the fluorescence of a thin sheet of antibody solution.

### Spreading experiments

Experiments were performed as previously described 24 in custom-made chambers made of pMHC-coated coverslips forming the floor (1 cm^2^) of teflon-walled wells containing 0.5 mL of HEPES-buffered RPMI medium supplemented with 10% FCS. They were maintained at 37°C in a heating enclosure (TRZ 3700, Zeiss) mounted on an inverted microscope. About 250 000 cells suspended in warm medium were added, and an observation field was selected for 10 min continual monitoring with video recording. A series of microscope field images were then acquired to determine the average contact area between 15 and 20 min after initial contact with better statistical accuracy. A total of 495 cells were thus followed for continual monitoring and a total of 13 574 instantaneous cell images were recorded. Data corresponding to a given condition (i.e., pMHC species + concentration) were a pool of four to six separate experiments.

### Image acquisition and processing

IRM was performed as previously described 24,26. Briefly, cells were examined with an Axiovert 135 inverted microscope (Zeiss, Germany) using a 63× Antiflex^TM^ objective and 546 nm excitation wavelength. Images were obtained with an Orca C4742-95-10 camera (Hamamatsu, Japan) as stacks of typically 500–700 images of 8-bit depth and 1024 × 1024 pixel size captured with 1 Hz frequency for each monitored cell. Pixel size was 125 × 125 nm^2^. Images were corrected by mean filtering and linear compensation for variations of background intensity. Cell/substratum distance *d* at each pixel was derived from illumination intensity *I* with the low incidence approximation 26,40:

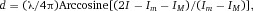
2where λ is the light wavelength in aqueous medium, and *I_m_* and *I_M_* are, respectively, the minimum and maximum intensities corresponding to *d* = 0 and *d* = λ/4 ≈ 100 nm, respectively. All calculations were performed with a custom-made image processing software written in C++ 30. Molecular contact between cells and surfaces was assumed to occur when the calculated distance *d* was ≤34 nm on at least two pixels.

Initial spreading was defined as the first time point where contact was initiated and (i) contact was maintained for at least 45 s, and (ii) contact area reached a minimum level of 7.8 μm^2^ (corresponding to 500 pixels). The maximum spreading rate was obtained by calculating the maximum average spreading rate during a 45-s period from the initial spreading to the end of the observation period.

### Statistics

ANOVA was performed with Satterwaith’s correction for unequal samples 41.

## Abbreviations

IRM: interference reflection microscopy
pMHC: oligopeptide–MHC

## Supporting Information

As a service to our authors and readers, this journal provides supporting information supplied by the authors. Such materials are peer reviewed and may be re-organized for online delivery, but are not copy-edited or typeset. Technical support issues arising from supporting information (other than missing files) should be addressed to the authors.

## References

[b1] Altan-Bonnet G, Germain RN (2005). Modeling T cell antigen discrimination based on feedback control of digital ERK responses. Plos Biol.

[b2] Sykulev Y, Joo M, Vturina I, Tsomides TJ, Eisen HN (1996). Evidence that a single peptide-MHC complex on a target cell can elicit a cytolytic T cell response. Immunity.

[b3] Irvine DJ, Purbhoo MA, Krogsgaard M, Davis MM (2002). Direct observation of ligand recognition by T cells. Nature.

[b4] Huang J, Brameshuber M, Zeng X, Xie J, Li Q-J, Chien Y-H, Valitutti S (2013). A single peptide-major histocompatibility complex ligand triggers digital cytokine secretion in CD4+ T cells. Immunity.

[b5] Huse M, Klein LO, Girvin AT, Faraj JM, Li Q-J, Kuhns MS, Davis MM (2007). Spatial and temporal dynamics of T cell receptor signaling with a photoactivatable agonist. Immunity.

[b6] Dustin ML, Bromley SK, Kan Z, Peterson DA, Unanue ER (1997). Antigen receptor engagement delivers a stop signal to migrating T lymphocytes. Proc. Natl. Acad. Sci. USA.

[b7] Bunnell SC, Kapoor V, Trible RP, Zhang W, Samelson LE (2001). Dynamic actin polymerization drives T cell receptor-induced spreading: a role for the signal transduction adaptor LAT. Immunity.

[b8] Francois P, Voisinne G, Siggia ED, Altan-Bonnet G, Vergassola M (2013). Phenotypic model for early T-cell activation displaying sensitivity, specificity and antagonism. Proc. Natl. Acad. Sci. USA.

[b9] Dushek O, van der Merwe PA (2014). An induced rebinding model of antigen discrimination. Trends Immunol.

[b10] Matsui K, Boniface JJ, Steffner P, Reay PA, Davis MM (1994). Kinetics of T-cell receptor binding to peptide/I-Ek complexes: correlation of the dissociation rate with T-cell responsiveness. Proc. Natl. Acad. Sci. USA.

[b11] Alam SM, Travers PJ, Wung JL, Nasholds W, Redpath S, Jameson SC, Gascoigne NRJ (2000). T-cell receptor affinity and thymocyte positive selection. Nature.

[b12] Kalergis AM, Boucheron N, Doucey MA, Palmieri E, Goyarts EC, Vegh Z, Luescher IF (2001). Efficient T cell activation requires an optimal dwell-time of interaction during the TCR and the pMHC complex. Nat. Immunol.

[b13] Aleksic M, Dushek O, Zhang H, Shenderov E, Chen J-L, Cerundolo V, Coombs D (2010). Dependence of T cell antigen recognition on T cell receptor-peptide MHC confinement time. Immunity.

[b14] Dushek O, Aleksic M, Wheeler RJ, Zhang H, Cordoba S-P, Peng Y-C, Chen J-L (2011). Antigen potency and maximal efficacy reveal a mechanism of efficient T cell activation. Sci. Signal.

[b15] Robert P, Aleksic M, Dushek O, Cerundolo V, Bongrand P, van der Merwe PA (2012). Kinetics and mechanics of two-dimensional interactions between T cell receptors and different activating ligands. Biophys. J.

[b16] Huang J, Zarnitsyna VI, Liu B, Edwards EJ, Jiang N, Evavold BD, Zhu C (2010). The kinetics of two-dimensional TCR and pMHC interactions determine T-cell responsiveness. Nature.

[b17] Huppa JB, Axmann M, Mörtelmaier MPA, Lillemeier BF, Newell M, Brameshuber EW, Klein (2010). TCR-peptide-MHC interactions in situ show accelerated kinetics and increased affinity. Nature.

[b18] Liu B, Chen BD, Evavold W, Zhu C (2014). Accumulation of dynamic catch bonds between TCR and agonist peptide-MHC triggers T cell signaling. Cell.

[b19] Hopfield JJ (1974). Kinetic proofreading: a new mechanism for reducing errors in biosynthetic processes requiring high specificity. Proc. Natl. Acad. Sci. USA.

[b20] McKeithan TW (1995). Kinetic proofreading in T-cell receptor signal transduction. Proc. Natl. Acad. Sci. USA.

[b21] Dushek O, Das R, Coombs D (2009). A role for rebinding in rapid and reliable T cell responses to antigen. PLoS Comput. Biol.

[b22] He HT, Bongrand P (2012). Membrane dynamics shape TCR-generated signalling. Front. Immunol.

[b23] Klotzsch E, Schütz GJ (2013). Improved ligand discrimination by force-induced unbinding of the T cell receptor from peptide-MHC. Biophys. J.

[b24] Crétel E, Touchard D, Bongrand P, Pierres A (2011). A new method for rapid detection of T lymphocyte decision to proliferate after encountering activating surfaces. J. Immunol. Methods.

[b25] Brodovitch A, Bongrand P, Pierres A (2013). T lymphocytes sense antigens within seconds and make a decision within one minute. J. Immunol.

[b26] Pierres A, Eymeric P, Baloche E, Touchard D, Benoliel A-M, Bongrand P (2003). Cell membrane alignment along adhesive surfaces: contribution of active and passive cell processes. Biophys. J.

[b27] Foa C, Mège JL, Capo C, Benoliel A-M, Galindo J-R, Bongrand P (1988). T-cell-mediated cytolysis: analysis of killer and target cell deformability and deformation during conjugate formation. J. Cell Sci.

[b28] Limozin L, Sengupta K (2009). Quantitative reflection interference contrast microscopy (RICM) in soft matter and cell adhesion. ChemPhysChem.

[b29] Theodoly O, Huang ZH, Valignat MP (2010). New modeling of reflection interference contrast microscopy including polarizatin and numerical aperture effects: application to nanometric distance measurements and object profile reconstruction. Langmuir.

[b30] Pierres A, Benoliel A-M, Touchard D, Bongrand P (2008). How cells tiptoe on adhesive surfaces before sticking. Biophys. J.

[b31] Viola A, Lanzavecchia A (1996). T cell activation determined by T cell receptor number and tunable thresholds. Science.

[b32] Palmer E, Naeher D (2009). Affinity threshold for thymic selection through a T-cell receptor-co-receptor zipper. Nat. Rev. Immunol.

[b33] Sage PT, Varghese LM, Martinellli R, Sciuto TE, Kameil M, Dvorak AM, Springer TA (2012). Antigen recognition is facilitated by invadosome-like protrusions formed by memory/effector T cells. J. Immunol.

[b34] Bentley D, Toroian-Raymond A (1986). Disoriented pathfinding by pioneer neurone growth cones deprived of filopodia by cytochalasin treatment. Nature.

[b35] Faix J, Rottner K (2006). The making of filopodia. Curr. Opin. Cell Biol.

[b36] Tay S, Hughey JJ, Lee TK, Lipniacki T, Quake SR, Covert MW (2010). Single-cell NF-κB dynamics reveal digital activation and analogue information processing. Nature.

[b37] Bousso P, Bhakta NR, Lewis RS, Robey E (2002). Dynamics of thymocyte-stromal cell interactions visualized by two-photon microscopy. Science.

[b38] Miller MJ, Wei SH, Parker I, Cahalan MD (2002). Two-photon imagin of lymphocyte motility and antigen response in intact lymph nodes. Science.

[b39] Vitte J, Benoliel AM, Eymeric P, Bongrand P, Pierres A β-1 integrin-mediated adhesion may be initiated by multiple incomplete bonds, thus accounting for the functional importance of receptor clustering. Biophys. J.

[b40] Zidovska A, Sackmann E (2006). Brownian motion of nucleated cell envelopes impedes adhesion. Phys. Rev. Lett.

[b41] Snedecor GW, Cochran WG (1980). Statistical methods.

